# Terrell Medical Society—Abstract of Proceedings

**Published:** 1890-06

**Authors:** E. E. Smiley

**Affiliations:** Secretary


### Society Notes.

### TERRELL MEDICAL SOCIETY—ABSTRACT OF PROCEEDINGS.

This flourishing society holds its meetings twice a month; 1st and 3rd Mondays. President, Dr. J. A. Anthony; Secretary, Dr. E. E. Smiley.

Paper on "Rötheln," by Dr. Nelson; held that it is a distinctive disease; but sometimes associated with measles and scarletina, and often mistaken for the latter. Relapses are frequent, with arrest of renal secretion, and is rebellious to treatment.

Dr. Stroud reported a case of jaundice, in which there was complete suppression of urine; yielded to hot baths, fl. ex. digitalis and potas bi-tart. Speaking of jaundice, Dr. Inabnit said "black jaundice is a misnomer and conveys no idea of the pathology of the disease; malignant jaundice is the proper name." [Malarial hæmaturia?—Ed.]

Dr. Nelson asked what had been the experience of members this season in the use of quinine in the fevers of the country? Drs. Inabnit, Anthony and Jones had not found it as satifactory as in former years. Drs. Dumas and Strain had seen no difference.

Dr. Dumas reported a case of gun-shot wound of the liver; ball entered to right of and below the enciform cartilege; ball not found; subsequent fluctuation showed the presence of pus,—aspirated and evacuated a large quantity of pus. Patient recovered and experienced no inconvenience from the presence of the ball.

Dr. Smiley reported a case of erysipelas of lower limb treated successfully with campho phenique locally. Gave but little internal treatment, as he desired to test the value of this new remedy. Dr. Fry said, this success with local treatment overthrows the theory of blood poison,—which, necssarily, creates a constitutional trouble, and he could not depend alone on any local treatment. Dr. Nelson thought that in the lighter cases and superficial, local treatment could be relied upon.

Dr. Strain amputated the leg in upper third as a *dernier ressort* in a case of gangrene from blood poison; patient much emaciated, and death without the operation certain. Patient recovered.

For carbuncle Dr. White recommended carbolic acid and cocaine injected into the tumor. Dr. Orr, in a paper on the "germ theory", held that the "germs" are the result and not the cause of the disease.

Dr. Orr has found that of late years gonorrhœa is more difficult to cure than formerly; the remedies, formerly successful, now produced little or no satifactory results. Dr. Inabnit recommended him to try 40 grs. [?] of chloral hydrate to the ounce of water as an injection; he had found it eminently satisfactory.

For an obstinate case of parotitis Dr. Smiley recommended local application of fluid ext. phytolacca as a stimulating absorbant to the glandular system. Dr. Orr preferred muriate ammonia sc. iii to the ounce of water.

Dr. Sanders called attention to the great value of gelsemium internally in intermittent fevers, in phthisis, and as a uterine laxative [?], and claimed that it is also the best application for rhus-toxicodendron-poisoning—one application being sufficient generally.

Dr. L. M. Stroud read an essay on abdominal surgery, and dwelt upon the advantage of uniting severed intestines by means of the decalcified bone plate.

A new use for iodide of potas. Dr. Smiley said, was to prescribe two grains to half a glass of water for frontal headache. The patient must take the whole, slowly with a teaspoon at bedtime. Next morning the patient will be seized with a paroxysm of sneezing and will discharge large quantities of muco-pus [?] from the nose, with immediate relief. Quinine and Dovers powder will check excessive action.

Dr. Tidwell exhibited a young man, who, from jumping, had become paralized. He recovered under electricity.

Dr. Dumas exhibited a drawing of an hour glass contraction, and an interesting discussion followed—as to what caused the contraction; ergot being accused by some as being the prime cause.

Dr. F. S. White read two papers,—one giving an account of suicide by a melancholy inmate of the lunatic asylum, and the other decribing a malformation or arrest of development in the genitalia of a patient who had died in the asylum,—a case of so-called hermaphroditism.*

E. E. SMILEY, Secretary.

[Much interest is taken in the work of this society, and the meetings are largely attended. It should be a stimulant to other local societies.—ED.]

---

[*These papers were voted to be published in DANIEL'S TEXAS MEDICAL JOURNAL, and will appear in due time.—ED.]

